# Targets of the *Entamoeba histolytica* Transcription Factor URE3-BP

**DOI:** 10.1371/journal.pntd.0000282

**Published:** 2008-08-27

**Authors:** Carol A. Gilchrist, Duza J. Baba, Yan Zhang, Oswald Crasta, Clive Evans, Elisabet Caler, Bruno W. S. Sobral, Christina B. Bousquet, Megan Leo, Ameilia Hochreiter, Sarah K. Connell, Barbara J. Mann, William A. Petri

**Affiliations:** 1 Department of Medicine, University of Virginia, Charlottesville, Virginia, United States of America; 2 Virginia Bioinformatics Institute, Blacksburg, Virginia, United States of America; 3 J. Craig Venter Institute, Rockville, Maryland, United States of America; 4 Department of Microbiology, University of Virginia, Charlottesville, Virginia, United States of America; 5 Department of Pathology, University of Virginia, Charlottesville, Virginia, United States of America; Boston University, United States of America

## Abstract

The *Entamoeba histolytica* transcription factor Upstream Regulatory Element 3-Binding Protein (URE3-BP) is a calcium-responsive regulator of two *E. histolytica* virulence genes, *hgl*5 and *fdx*1. URE3-BP was previously identified by a yeast one-hybrid screen of *E. histolytica* proteins capable of binding to the sequence TATTCTATT (Upstream Regulatory Element 3 (URE3)) in the promoter regions of *hgl*5 and *fdx*1. In this work, precise definition of the consensus URE3 element was performed by electrophoretic mobility shift assays (EMSA) using base-substituted oligonucleotides, and the consensus motif validated using episomal reporter constructs. Transcriptome profiling of a strain induced to produce a dominant-positive URE3-BP was then used to identify additional genes regulated by URE3-BP. Fifty modulated transcripts were identified, and of these the EMSA defined motif T[atg]T[tc][cg]T[at][tgc][tg] was found in over half of the promoters (54% p<0.0001). Fifteen of the URE3-BP regulated genes were potential membrane proteins, suggesting that one function of URE3-BP is to remodel the surface of *E. histolytica* in response to a calcium signal. Induction of URE3-BP leads to an increase in tranwell migration, suggesting a possible role in the regulation of cellular motility.

## Author Summary

Most infections with *Entamoeba histolytica* are asymptomatic. However, in a minority of cases, they develop into invasive and even life-threatening amebiasis. We suspect, based on prior studies of invasive amebae, that changes in amebic gene expression enable the transition from asymptomatic to invasive infection. Our long-term goal is to identify the genetic program required to cause amebic colitis. Here, we studied a transcription factor named URE3-BP that controls the expression of two virulence genes, the Galactose and Galactose N- acetyl- galactosamine inhibitable lectin (Gal/GalNAc lectin) and ferredoxin. We suspected that this factor might coordinate invasiveness by co-regulating additional virulence factors. The consensus DNA motif that is recognized by URE3-BP was identified by reporter gene assays and by electromobility shift assays. We then inducibly expressed a constitutively active form of the transcription factor, and measured the changes in total amebic gene expression mediated by overexpression of this dominant-positive version of URE3-BP. This analysis allowed for a further definition of the functional URE3 motif. Inducible expression of URE3-BP led to changes in the transcript levels of several novel amebic membrane proteins. In conclusion, this genome-wide analysis of a transcription factor and its cis-acting regulatory sequence in *Entamoeba histolytica* has identified new transcripts regulated by URE3-BP that may play a role in trophozoite motility within a coordinated virulence-specific gene regulatory network.

## Introduction

The early branching eukaryote *Entamoeba histolytica* is a human parasite that is the etiologic agent of amebic dysentery and liver abscess. Only one of every five infections leads to disease [1], and the parasite and host factors that control the outcome of infection are not well understood. Alteration in transcription of certain crucial genes may contribute to the expression of a virulence phenotype. Distinct gene expression profiles which may be associated with pathogenicity have been identified by comparing the transcriptome of laboratory-cultured HM-1:IMSS *E. histolytica* to trophozoites growing *in vivo*, as well as to that of less virulent strains and recent clinical isolates [2],[3],[4],[5],[6].

Here we have attempted to study the molecular mechanisms involved in the transcriptional regulation of virulence in *E. histolytica* by investigating further the role of the upstream regulatory element 3-binding protein (URE3-BP) transcription factor.

URE3-BP is a calcium regulated transcription factor, that is known to bind to the URE3 motif and thereby modulate transcription of both the Gal/GalNAc-inhibitable lectin *hgl*5 and ferredoxin 1 (*fdx*) genes. Mutation of the URE3 motif within the *hgl*5 and *fdx*1 promoter led to a four-fold rise and a two-fold drop in gene expression respectively, indicating that URE3 may function as a repressor or activator depending on context [7],[8].

Previously a yeast one hybrid screen was used to identify an *E. histolytica* cDNA encoding a protein (URE3-BP) that recognized the URE3 DNA motif [9]. The URE3-BP protein was present in the *E. histolytica* nucleus and cytoplasm with an apparent molecular mass of 22.6 KDa. Two EF-hand motifs were identified in the amino acid sequence of URE3-BP. Binding of URE3-BP to the URE3 motif was inhibited *in vitro* by addition of calcium. Mutation of the second EF hand motif in URE3-BP resulted in the loss of calcium inhibition of DNA binding, as monitored by an electrophoretic mobility shift assay. Chromatin immunoprecipitation experiments confirmed the calcium-dependent interaction of URE3-BP with both the *hgl*5 and *fdx*1 promoter DNA [10].

Because the Gal/GalNAc inhibitable lectin is an important virulence factor of *E. histolytica* it may be coordinately regulated at the transcription level with other virulence genes. In this light, it was intriguing that the mRNA of (URE3-BP) was down regulated two-fold *in vivo* [2]. The discovery of direct downstream targets of URE3-BP therefore may identify other genes important in *E. histolytica* pathogenesis and help delineate molecular and cellular mechanisms involved in the expression of virulence.

Position-specific variability in the sequence of transcription factor binding sites renders recognition of valid targets by computational methods alone extremely challenging [11],[12] . Most work has been performed in the yeast model organism or the well-studied human transcriptome. The parameters affecting transcription regulation in early branching eukaryotes are only beginning to be deciphered [4],[13],[14],[15],[16],[17].

The sequencing of the *E. histolytica* genome identified homologues of most of the RNA polymerase II subunits [18],[19], however the structure of *E. histolytica* core promoter varies from the conventional norm by containing a third regulatory sequence GAAC in addition to the TATA box and INR. This may have an unpredictable impact on the machinery necessary for regulation of transcription [7],[20]. A bioinformatics approach was used by Hackney *et al* to correlate potential *E. histolytica* DNA motifs with high and low gene expression [21]. In our study we have focused on using not only computational but also experimental approaches to discover the gene regulatory network of the URE3-BP transcription factor.

To identify the consensus binding site sequence, a position weight matrix (PWM) of transcription factor binding to the URE3 motif was developed. To test the validity of the matrix, selected mutants within the URE3 motif of the *hgl*5 promoter were assessed for promoter activity in an episomal reporter construct. Finally, to identify additional genes regulated by URE3-BP, genome-wide expression profiling of transcripts from strains over-expressing a calcium insensitive URE3-BP mutant was performed.

## Methods

### Cultivation of *E. histolytica* and Nuclear Extract Preparation

*E. histolytica* strain HM1:IMSS trophozoites were grown at 37°C in TYI-S-33 medium containing penicillin (100 U/ml) and streptomycin (100 μg/ml) (GIBCO/BRL)[22]. Amebae in logarithmic phase growth (∼6×10^4^ trophozoites/ml) were used for nuclear extract preparation. Crude nuclear extracts were prepared by the method previously described [8],[23] with the following modifications: the protease inhibitors 2 mM (2S,3S)-trans-epoxysuccinyl-L-leucylamido-3-methylbutane and 2 mM 4-(2-aminoethyl) benzenesulfonylfluoride, HCl were added to both cell and nuclear lysis buffers, and dithiothreitol was omitted from the nuclear lysis buffer.

### Transient and stable transfection of *E. histolytica* trophozoites

Stable transfection of *E. histolytica* trophozoites was achieved by use of the previously described lipofection technique [24],[25] . Briefly, amebae were washed and suspended (2.2×10^5^ amebae per ml) in Medium 199 (Invitrogen, CA) supplemented with 5.7 mM cysteine, 1 mM ascorbic acid, 25 mM HEPES pH 6.8 (M199s) 3 μg of DNA and 15 μl of Superfect (Qiagen) was added. Treated amebae were left for 3 hours at 37°C, then growth media was added, and incubation at 37°C was continued overnight. The expression of all the recombinant proteins was confirmed by western blotting. Nuclear and cytoplasmic extracts were prepared using standard techniques [9]. Transfected amebae were selected with either G418 (6 μg/ml) or hygromycin (15 μg/ml). Transient transfection was achieved using the electroporation protocol described by Purdy *et al.* Briefly trophozoites were washed and suspended in 120 mM KCI, 0.15 mM CaCl_2_, 10 mM K_2_HPO4/KH_2_PO4, pH 7.5, 25 mM HEPES, 2 mM EGTA, 5 mM MgC1_2_, 50 μg/ml of plasmid and 3.1 μg/ml of DEAE-dextran, and electroporated at 500 μF and 500 V/cm (Gene Pulser, Bio-Rad) [7].

### Electrophoretic Mobility Shift Analysis

URE3-BP, has been shown to bind specifically to the TATTCTATT (URE3) DNA motif in Gilchrist *et al* 2001 [9]. In these conditions antibodies raised against URE3-BP blocked the formation of the URE3 DNA-protein complex by native nuclear extracts and competition with a 60 fold excess of the nonspecific oligonucleotide (Olig-1) did not interfere with the formation of the specific complex. EMSA assays were performed with a Klenow-radiolabeled double stranded DNA oligonucleotide that spans the URE3 motif within the *hgl*5 promoter TGTTCCAAAAAGATATATTCTATTGAAAATAAAAGAAG (*hgl*5-URE3). The protein-DNA interaction occurred in band shift buffer (10 mM Tris-HCl [pH 7.9], 50 mM NaCl, 1 mM EDTA, 0.05% nonfat milk powder, 3% glycerol, 0.05 mg of bromophenol blue) to which 0.2 μg of poly(dIdC), 10 fmol of DNA probe, and 2 μg of nuclear extract were added. The reaction mixture was allowed to incubate at room temperature (20°C) for 1 h prior to electrophoresis on a nondenaturing polyacrylamide gel for 2 to 3 h. The gel was then fixed and dried, and the signal from the protein-DNA complex was quantitated after exposure of the gel to a phosphorimage screen as described previously [8]. A ten fold or six fold excess of either cold hgl5-URE3 (wt) or oligonucleotides wherein a base pair alteration within the URE3 motif had been made were added to the assay and the amount of competition was quantitated using a PhosphorImager. A double stranded oligonucleotide (Olig1) with the sequence AGAAAGCGTAATAGCTCA was used as an irrelevant control. Experiments were performed in triplicate, gels scanned (Molecular Dynamics, Model 425) and relative density of the EMSA assessed by use of the ImageQuant program (IQMac v1).

### Stable and Inducible expression vectors

The stable construct (pHTP.luc) contained the luciferase structural gene under the control of the *E. histolytica hgl*5 gene [26]. The promoter was mutated at the URE3 motif as described in results. Inducible vectors were based on the tetracycline inducible gene expression system of Ramakrishnan *et al*. [27]. An N-terminal myc tag was introduced by the amplification using the oligonucleotide TGCGGATCCAAATGGAACAAAAATTAATTTCAGAAGAAGATTTA-ATGCAACCACCTGTAGCTAATTTCC, and a control generated using an oligonucleotide that incorporated two stop codons directly after the myc tag (CTTGTATTTAACAATAGCTAACATC). Both amplicons were subcloned into the pCR2.1 TOPO expression vector (Invitrogen) and sequenced to confirm the presence of the desired mutations. The DNAs were then subcloned into the tetracycline-inducible gene expression system.

### RNA isolation

One ml of Trizol (Invitrogen) was added to 2×10^6^ amebae collected by centrifugation at 900 rpm for 5 min and an initial RNA preparation performed according to the manufacturer's directions. RNA greater than 200 nucleotides in length was separated from total RNA by the RNeasy protocol (Qiagen). RNA was isolated from at least two independent cultures on the same day for microarray analysis.

### qRT-PCR

Reverse transcription real time PCR (qRT-PCR) was used to independently measure mRNA abundance in independently transformed amebae. The cDNA was subjected to 40 amplification cycles with HotStarTaq (Qiagen). Primers were designed to amplify 100–300 base pairs using genomic sequences from the *E. histolytica* Genome Sequencing Project (http://www.tigr.org/tdb/e2k1/eha1/, http://pathema.tigr.org/tigr-scripts/Entamoeba/PathemaHomePage.cgi) and the Primer3 program (Table S1) [28]. The fluorescent dye SYBR Green I (Molecular Probes) was used to detect amplified cDNA. Continuous SYBR Green I monitoring during amplification using the MJR Opticon II machine was done according to the manufacturer's recommendations. All real time amplification reactions were performed in triplicate and the resulting fluorescent values averaged. In all experiments utilizing qRT-PCR the cycle threshold values (C_T_, the cycle number at which fluorescence exceeds the threshold value) were linked to the quantity of initial DNA after calibration of the effectiveness of the amplifying primer pair [29]. The relatively invariant *lgl*1 transcript was used to compensate for the variation in the amount of amebic mRNA isolated.

### Hybridization of sample to the Affymetrix E_his-1a520285 custom array

Quality control of RNA samples was performed by use of the Agilent Bioanalyser Nano Assay. The standard protocol for hybridization of eukaryotic mRNA to Affymetrix arrays was followed (http://www.affymetrix.com/support/technical/manual/expression_manual.affx). Two micrograms of total RNA was used for cDNA and subsequent biotinylated cRNA synthesis. This labeled RNA probe was hybridized to the Affymetrix custom array designed using information generated from the *E. histolytica* genome sequencing project release date 12/08/04 as previously described [2],[18]. The affymetrix probes were mapped to the new Genome Assembly and recognized 6385 of the reannotated open reading frames (78% of *E. histolytica* Open Reading Frames (ORF) 8197 http://pathema.tigr.org/). The ORF probe sets were preferentially selected from the 600 bases proximal to the 3′ end of the *E. histolytica* sequences. The arrays were scanned with an Affymetrix Gene Chip scanner 7G and report files were generated to determine the percentage of present calls of each array. The detection calls (present, marginal, absent) for each probe set were obtained using the GCOS system (http://www.affymetrix.com/products/software/specific/gcos.affx). Only genes with at least one “present” call were used in assessment of the data. Raw data from the arrays were normalized at probe level by the gcRMA algorithm and then log2 transformed [30].

*Genome analysis and datasets−* The dataset used in this analysis was that of the reannotated *E. histolytica* genome of Caler *et al*. (manuscript in preparation) publicly available at http://pathema.tigr.org (Genebank accession number (AAFB00000000)). The reannotated genome was searched for the URE3 motif with a custom motif search script (Table 1).

**Table 1:** Presence of the URE3 matrix in the promoters of genes modulated by a dominant positive URE3-BP

	Promoters which did not contain a URE3 matrix	Promoters containing a URE3 matrix 375-25 bases 5′ of ATG start codon	Total Number of Promoters analyzed	%	Statistical significance
Transcripts significantly modulated by URE3-BP	23	27	50	54	p<0.0001
All *E. histolytica* promoters	6527	1985	8522	23	
**Promoters with multiple URE3**					
	Promoters which did not contain at least 2 URE3 matrices				
Transcripts significantly modulated by URE3-BP	40	10	50	20	p = 0.0031
All *E. histolytica* promoters	7862	660	8522	8	
**Motif Frequency in Promoters**					
	Nonamers not in motif	URE3 motifs	potential motifs (sequence divided by motif length 9 bp)		
Transcripts significantly modulated by URE3-BP	1905	39	1944.4	2	p = 0.0025
All *E. histolytica* promoters	327360	4051	331411	1.2	

A contingency table χ^2^ test was used to compare the occurrence of the URE3 motif in transcripts significantly modulated by URE3 (the numbers were to large for the Fishers exact test). The motif background in all *E. histolytica* promoters was determined using a custom motif search script. To clearly illustrate the data we show the analysis on a promoter basis, and motif frequency per sequence nonamer.

### Statistical analysis

Microarray data analysis was performed using the Array Data Analysis and Management System (VBI) (http://pathport.vbi.vt.edu/main/microarray-tool.php). The system uses publicly available tools such as Bioconductor [31] for analysis of the data. Briefly, statistical significance was determined for the microarray data using the Linear Models for Microarray Data (LIMMA) program as described in the results section [32],[33]. The statistical significance p values were corrected using the Benjamini and Hochberg false-discovery-rate test (FDR≤0.05) [34]. Our comparisons were both between the two strains, and between different time points giving us potentially three control conditions. The most comprehensive comparison was between the test and control strains at 9 h post-induction. Statistical significance was determined for the qRT-PCR results using the students T test and the non-parametric Kruskal-Wallis Test was used to determine significance in the reporter gene assays. URE3 associated promoters were compared to the frequency of motif appearance in all *E. histolytica* promoters using the chi-squared test (InStat 2.03 program (GraphPad Software)).

### Transwell migration assays

Transwell migration assays were performed using 5 mm transwell inserts (8 μm pore size Costar) suspended by the outer rim within individual wells of 24-well plates. Briefly, ameba trophozoites were incubated in serum free growth media containing 2 μg/ml CellTracker Green CMFDA (Molecular Probes) for 1h [35]. Trophozoites were then washed and suspended at a concentration of 2×10^5^/ml in serum free media and 500 μl loaded into the upper chamber. The plates were then placed in anaerobic bags (GasPak 100 Anerobic system; BD Biosciences) and incubated at 37°C for 3 h. Inserts and media were removed and fluorescence measured using a SpectraMax M2 fluorescent plate reader. Fluorescence versus concentration for each sample was determined by using a standard curve. Ameba numbers confirmed in selected experiments by microscopic counting and by use of the Techlab *E. histolytica* II antigen test used according to the manufacturer's directions.

## Results

### URE3 Matrix

**Figure 1:**
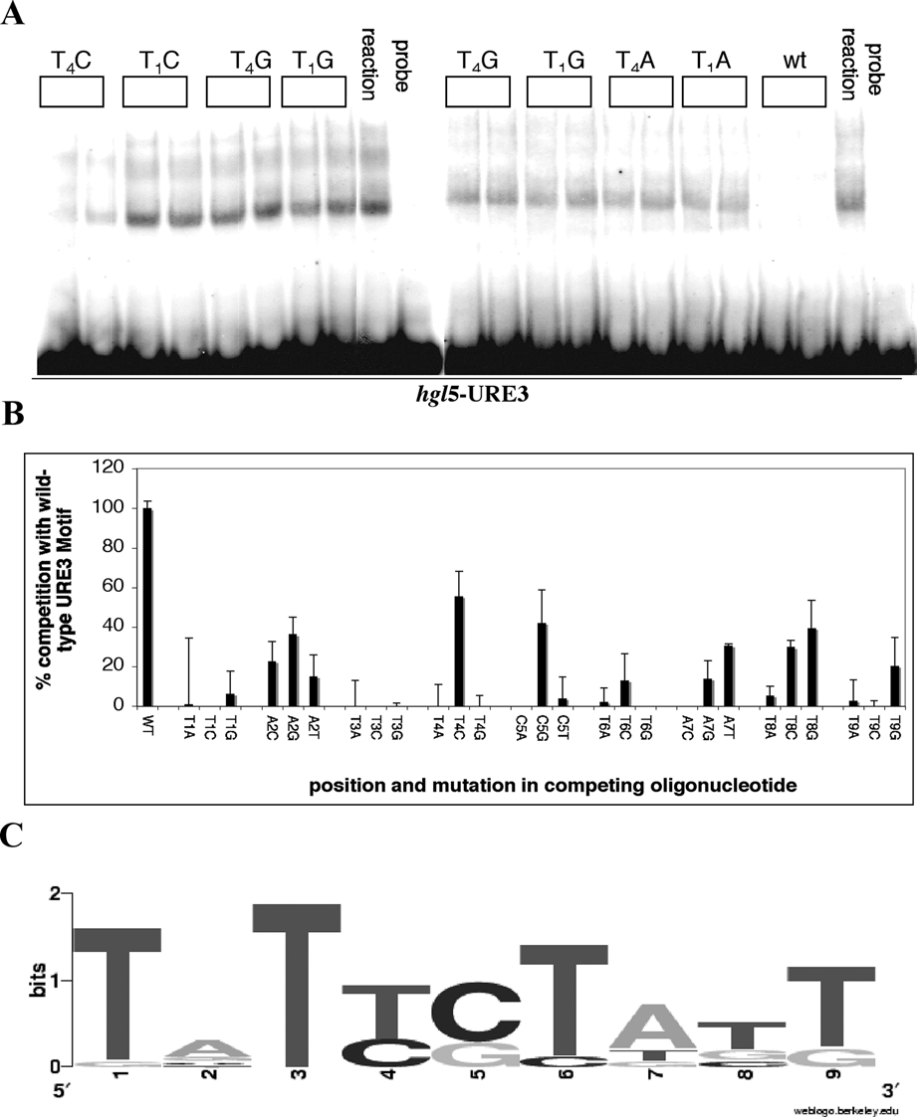
URE3 Matrix Discovery. (A) Representative electrophoretic mobility shift assay (EMSA) performed with radioactively labeled *hgl*5-URE3 double-stranded DNA. The lanes with probe alone are indicated; all other reactions included 2 μg of *E. histolytica* nuclear extract. EMSA's were performed with a Klenow-radiolabeled double stranded DNA oligonucleotide that spanned the URE3 motif within the *hgl*5 promoter TGTTCCAAAAAGATATATTCTATTGAAAATAAAAGAAG (*hgl*5-URE3). A ten fold excess of either cold hgl5-URE3 (wt), or an oligonucleotide with a base pair substitution within the URE3 motif, was used as a competition to the wild type oligonucleotide. These are indicated by position and base substitution (i.e. T_4_C indicates that the T at position 4 in the URE3 motif [TATT_4_CTATT] was changed to a C). The image was generated with a PhosphorImager (Molecular Dynamics model 425) in conjunction with the Adobe PhotoShop software program. (B) DNA-binding profile of URE3-BP derived from the EMSA results. The intensity of an irrelevant control was set as 100% and competition of the wild type oligonucleotide set as 0% (y axis). The position and base changes in the competing oligonucleotides T_1_A_2_T_3_T_4_C_5_T_6_A_7_T_8_T_9_ are shown on the x axis. The results of three independent replicates were averaged and are shown as a mean with standard error. (C) Graphical representation of the URE3 consensus sequence. The percent contribution of each base to the total competition occurring at each position (from each of the four bases) was calculated and shown graphically using the sequence logo program of Crooks *et al.*

Electrophoretic mobility shift analysis (EMSA) was used with base substituted oligonucleotides to define the consensus URE3 motif. The impact of adding an excess of a non-radioactive oligonucleotide with a base pair alteration within the URE3 motif T_1_A_2_T_3_T_4_C_5_T_6_A_7_T_8_T_9_. was measured. A representative gel showing competition with the motif modified at positions 1 (AATTCTATT, GATTCTATT, CATTCTATT) or 4 (TATACTATT, TATGCTATT, TATCCTATT) is shown in Figure 1A. The efficacy of a substituted base in competition assays was compared to the wild type motif (100%) and an irrelevant control (0%), as shown in Figure 1B. The percent contribution of each base to the total competition occurring at each position (from each of the four bases) was then calculated and is shown graphically in Figure 1C. The consensus URE3 motif incorporated base substitutions that maintained at least 15% competition of the gel shifts. The prototypic URE3 motif T_1_A_2_T_3_T_4_C_5_T_6_A_7_T_8_T_9_ as a result was modified to a consensus motif of T_1_[atg]_2_T_3_[tc]_4_[cg]_5_T_6_[at] _7_[tgc]_8_[tg]_9_.

### Verification of the matrix by reporter gene assays

**Figure 2:**
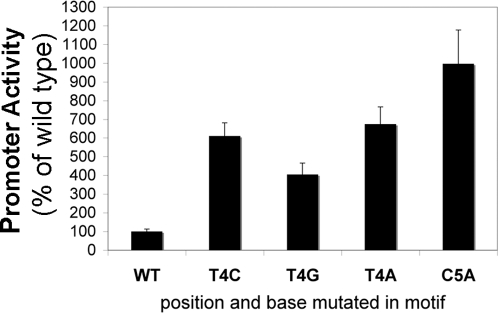
Transcriptional Activity of the URE3 Motif as Assessed by URE3-driven Repression of a Reporter Gene. The *hgl*5 promoter (wild type and containing the T_4_A, T_4_C, T_4_G and C_5_A mutations) was placed upstream of the luciferase reporter gene. These constructs were transfected into cultured *E. histolytica* trophozoites. Mutations (T_4_A and C_5_A) identical to those within the competing oligonucleotides were made within the *hgl*5 promoter URE3 motif. Luciferase values from at least three independent experiments with two different DNA preparations were performed. Luciferase values standardized to wt (100%) are shown as means with standard error. Promoter activity as a % of wild type is shown on the y axis and position and base mutated in the promoter on the x axis. All mutants were statistically different from the wild type promoter (p<0.0001 using the non-parametric Kruskal-Wallis Test).

We tested whether URE3 mutations that prevented competition in EMSAs (Figure 1), also blocked URE3 function in a transfected promoter. Key bases within the *hgl*5 promoter URE3 motif were mutated: T_4_A, T_4_C T_4_G and C_5_A. These mutant promoter sequences were placed upstream of the luciferase reporter gene. Luciferase values from at least three independent experiments with two different DNA preparations were performed (Figure 2). De-repression of the promoter in all base changes assayed indicated that these bases were critical for the binding of URE3-BP (which acts as a repressor in the *hgl*5 promoter context). This included the promoter with the mutation T_4_C. In the EMSA assay the T_4_C oligonucleotide affinity for URE3-BP was approximately 50% of the wild type oligonucleotide. We interpreted this as a consequence of the lower sensitivity of the episomal reporter assays, likely due to over-expression of episomal constructs.

### Microarray analysis

**Figure 3:**
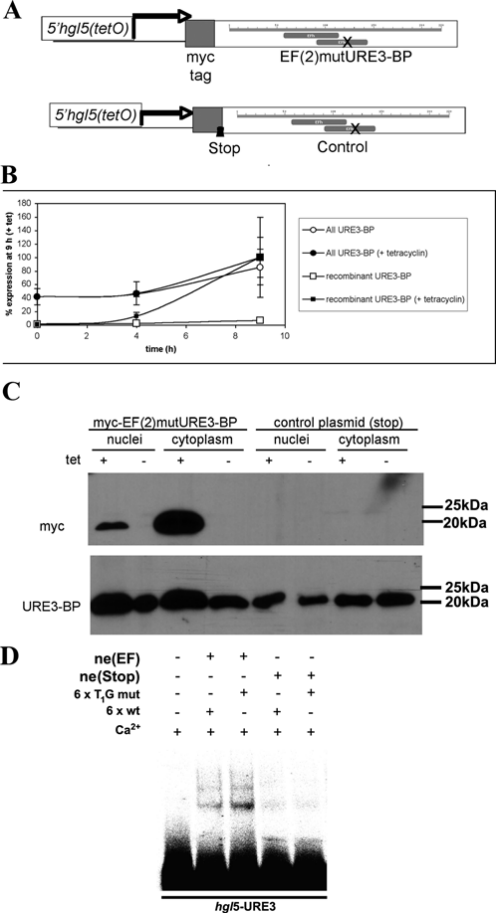
Inducible Overexpression of a Calcium-Insensitive Mutant of URE3-BP (EF(2)mutURE3-BP) in *E. histolytica*. A recombinant version of URE3-BP was generated by mutating one of the two EF-hand motifs of URE3-BP (associated with the ability to bind calcium, EF(2)mutURE3-BP [10]) and by introducing an N terminal myc tag. As calcium inhibited DNA binding by URE3-BP, this generated a dominant positive mutant [10]. The recombinant protein was placed under the control of a tetracycline-inducible gene expression system of *E. histolytica* (previously described by Ramakrishnan *et al.* [27]). As a control, in a second construct the initial N terminal sequence of URE3-BP was replaced by the sequence CTTGTATTTAACAATAGCTAACATC, mutated bases underlined, which introduced stop codons into the two open reading frames at the N terminus. (A) Cartoon showing the salient features of the constructs. (B) qRT-PCR of un-induced and induced ameba transfected with the pEF((2))mutURE3-BP. Results are normalized to the levels of *lgl*1 and shown as a percentage of values of ameba induced for 9 h (y axis). Time after induction is shown on the X axis. (C) Western blot of nuclear and cytoplasmic extracts from tetracycline-induced and un-induced amebae probed with an antibody specific for the myc tag and therefore the recombinant protein (9E10), as well as with a monoclonal antibody to URE3-BP (4D6). (D) Calcium insensitive binding to URE3 DNA in extract prepared from EF(2)mutURE3-BP transformed trophozoites. EMSA performed with added calcium and radioactively labeled *hgl*5-URE3 double-stranded DNA. Other than the lane with probe alone, reactions included 2 μg of *E. histolytica* nuclear extract prepared from either induced trophozoites carrying EF(2)mutURE3-BP or as a control STOP- EF(2)mutURE3-BP. A six fold excess of either cold hgl5-URE3 (wt), or an oligonucleotide with a base pair change which substituted a G for a T at the first position and had no impact on URE3-BP specific band formation (T_1_G mut) were added as shown.

To further evaluate the physiological relevance of the URE3 matrix, a calcium-insensitive mutant of URE3-BP (EF(2)mutURE3-BP) (Figure 3A), and therefore constitutively active, was inducibly expressed and the changes in gene expression measured by use of an Affymetrix custom array (E_his-1a520285).

The array included probes to 6,385 *E. histolytica* ORFs. Total RNA (12 μg) was isolated before induction (–Tet) and after 9 h of induction (+Tet) from cells carrying the myc-tagged recombinant URE3-BP mutant or the control construct (containing a stop codon immediately after the N terminal myc tag). The expression of the mRNA encoding the recombinant calcium-insensitive dominant positive mutant URE3-BP was induced 10–15 fold at nine hours post induction as indicated by myc specific qRT-PCR (Figure 3B). A western blot of *E. histolytica* nuclear and cytoplasmic proteins, probed with a myc-specific antibody, confirmed the cytosolic and nuclear distribution of both wild type and recombinant protein (Figure 3C). A calcium insensitive EMSA with *hgl*5-URE3 occurred only in nuclear extracts prepared from EF(2)mutURE3-BP transformed trophozoites (Figure 3D). In low calcium conditions EF(2)mutURE3-BP and STOP-EF(2)mutURE3-BP had equivalent URE3 binding capacity (data not shown).

### Statistical analysis of microarray data

**Figure 4:**
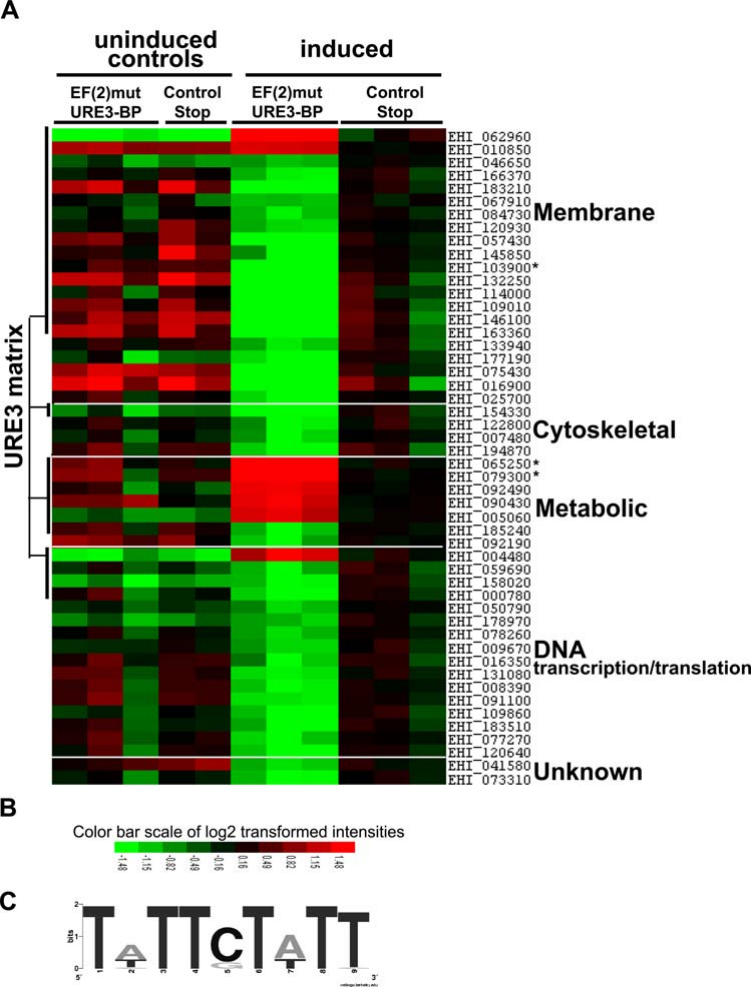
Comparison of *E. histolytica* Gene Expression upon Inducible Expression of EF(2)mutURE3-BP and STOP- EF(2)mutURE3-BP. (A) Heat map generated from microarray data reflecting gene expression values. Each column represents a microarray. Each row represents the expression pattern of one probe set across microarrays. The source of the RNA hybridized to each chip is indicated at the top of the columns. The ratios of transcript levels between experiments are color-coded in red and green. Red represents an increase of the transcript level of a gene in the transcript signal in this array compared to the expression of the transcript in induced STOP- EF(2)mutURE3-BP and green represents a decrease as indicated by the figure color bar. The genes modulated on induction of EF(2)mutURE3-BP, as compared to STOP- EF(2)mutURE3-BP are organized by functional category as shown in Table 2. Transcripts that had a potential URE3 matrix in the sequences −375-25bp 5′ of the start ATG codon, are indicated and detailed in Table 2 changes in transcript levels verified by qRT-PCR are marked by an asterisk (*). (B) Color bar scale of log2 transformed intensities. (C) Graphical representation of the observed URE3 sequences. The percent representation of the nucleotide occurring at each position (from each of the four bases) shown graphically using the sequence logo program of Crooks *et al* [53].

The complete microarray data (deposited in NCBI's Gene Expression Omnibus [36] and accessible through GEO Series accession number GSE12188 (http://www.ncbi.nlm.nih.gov/geo/query/acc.cgiaccGSE12188) was normalized using gcRMA and statistical significance determined by LIMMA statistical analysis (Table S2). A total of fifty mRNAs were increased or decreased ≥2-fold at 9h post-induction compared to the induced control strain in which an N-terminal stop codon was present in the EF(2)URE3-BP sequence (Figure 4). The filtered transcripts had a normalized signal intensity of >50 in at least one microarray experiment, a change of greater than 2 fold, and were statistically significant by LIMMA.

### Analysis of modulated transcripts

A total of fifty mRNAs were increased (8) or decreased (42) ≥2-fold at 9h post-induction compared to the induced “control stop” strain, which had a stop codon inserted after the sequence encoding the myc tag. To identify the novel URE3-BP regulated genes, the promoters of transcripts significantly modulated by two-fold or greater were scored for the presence or absence of the URE3 matrix. The DNA Pattern Find program (http://bioinformatics.org/sms/) was used to locate the URE3 matrix in putative promoters of URE3-BP responsive genes (Figure 4 and Table 2). In cases where the probe set represented a ‘family’ of highly similar transcripts the probe set was scored positive if any of the promoters contained a URE3 motif. The three family probe sets are indicated in Table 2.

**Table 2:** URE3 motif in promoters of genes modulated by a dominant positive URE3-BP

Pathema Loci ID	Annotation	Fold Change	Position of URE3 5′ of ATG	Location of Other URE3 motifs	Motif	Orientation^1^
**Proteins With Signal Peptides**						
EHI_062960	membrane protein, receptor-mediated transport^2,3^	4.19	133	124	TATTCTtTT	−
EHI_010850	EhCP-A2 &7	2.38	275		TATTCTATT	+
EHI_046650	membrane protein, similar to Gal/GalNAc lectin heavy subunit^4^	−2.01	134	113	Tt/gTTCTATT	−
				139		
EHI_166370	membrane protein	−2.07	182	35	TATTCTATT/g	−
EHI_183210	membrane protein^4,5^	−2.07	209		TtTTCTATT	−
EHI_067910	Competence protein ComEC^4^	−2.16	113		TATTCTtTT	+
EHI_084730	multidrug resistance-associated protein^2^	−2.28	147		TATTCTtTT	+
EHI_120930	transmembrane protein kinase^4^	−2.28	125	140	TATTC/gTATT	+
EHI_057430	membrane protein, surface antigen ariel1 family^6^	−2.47	46		TgTTCTATT	−
EHI_145850	membrane protein	−2.61	125		TATTGTATT	−
EHI_103900	membrane protein, nucleosome-binding protein 1	−3.3	46	127	TA/tTTGTATT	−
EHI_132250	membrane protein	−3.53	118		TATTGTATT	−
EHI_114000	membrane protein	−5.26	46	127	TA/tTTGTATT	−
EHI_109010	membrane protein^6^	−5.67	46		TtTTCTATT	−
EHI_146100	membrane protein	−6.11	46		TtTTCTATT	−
EHI_163360	membrane protein^6^	−6.56	46		TATTGTATT	−
**Cytoskeletal associated proteins**						
EHI_154330	Calponin homology domain^2^	−3.38	261	343	TATTCTtTT	−
**Metabolic enzymes**						
EHI_065250	membrane protein, Lecithin:cholesterol acyltransferase	5.25	106		TATTCTtTT	−
EHI_079300	acyl-CoA synthetase	2.98	362		TATTCTATT	+
EHI_092490	Protein with a weak similarity to sulfotransferases^2,7^	2.58	78	217	TATTC/gTT/tTT	+
EHI_090430	Protein with a weak similarity to sulfotransferase^7,8^	2.52	223		TtTTCTATT	−
EHI_005060	Fe-hydrogenase^9^	2.35	140		TtTTCTATT	−
EHI_185240	long-chain-fatty-acid--CoA ligase	−2.43	157	193	TtTTCTATT	−
**DNA transcription/translation**						
EHI_004480	basic leucine zipper protein^3^	2.36	80	89	TA/tTTCTATT	−
EHI_059690	chromosome segregation COG 1196	−2.33	51		TATTCTtTT	−
EHI_158020	transcription initiation factor IIIB chain BRF	−2.57	35		TATTCTtTT	−
EHI_000780	chromodomain-helicase-DNA-binding protein^4^	−2.94	128		TATTCTtTT	−

Identity (Pathema Locus number) and gene annotation of significantly modulated genes are shown in conjunction with the observed change between induced EF(2)mutURE3-BP and STOP- EF(2)mutURE3-BP strains. The distance between the URE3 motif and the presumed initiating ATG codon is shown as is the position of other potential URE3 motifs, the promoter consensus URE3 motif, and orientation.

1 If the motif is present in the promoter in a 5′ to 3′ direction this is indicated by (+) and on the reverse strand by (−).

2 These transcripts were decreased in recent clinical isolates [4]

3 Was modestly induced by tetracyclin at below threshold values

4 These transcripts were increased in recent clinical isolates [4]

5 This probe set recognizes a gene shown to be up regulated in HM-1:IMSS compared to a Rahman strain [3]

6 A signal peptide was found in either the 5′ extended open reading frame or 3′ of initiating ATG, the position of URE3 motif is shown from the newly designated initiating ATG

7 Annotation on the basis of homology to known sulfotransferases

8 This transcript was also significantly increased in the EF(2)mutURE3-BPuninduced ameba compared to the uninduced control.

9 This gene was described by Nixon *et al* and has been shown to be up regulated in HM-1:IMSS compared to a Rahman strain [3],[45]

The URE3 matrix was found in 23% of all predicted promoter regions, however the matrix appeared at a statistically greater frequency (54%) in the URE3-BP modulated transcripts predicted by LIMMA (chi-square test p>0.0001). Alternative analysis using the motif frequency or requiring the presence of two or more motifs for the positive designation also confirmed the correlation between the URE3 motif and transcript modulation (Table 1). The presence of the URE3 motif in the 3′ UTR regions was not above background values. The breakdown of the motifs found in the promoters of putative URE3-BP targets is shown in Table 2 and a graphical representation of the observed URE3 motifs is shown in Figure 4C. The sequence consensus of the URE3 motifs found 5′ of the modulated transcripts displayed only A or T residues at motif positions 2 and 7. While positions 2 and 7 were found to be the least conserved positions in the URE matrix consensus the predominant substitution of A/T may be a reflection of the AT bias of the *Entamoeba* genome. The other predominant change was a G substitution at position five which was half as effective as the wild type motif in EMSA assays (C_5_G).

InterPro was used to scan the open reading frames of the significantly modulated genes to obtain additional information on protein function, TMpred to predict transmembrane regions, big-PI Predictor to identify Glycosylphosphatidylinisotol (GPI) anchored proteins (GPI-anchor) and SignalP to identify signal peptides [37],[38],[39]. Sequences 150 bp 5′ and 3′ of the annotated ATG start codons were also checked and any additional in-frame peptides also examined for the presence of a signal peptide. On the basis of this information, the majority of the transcripts (47 of 50) could be subdivided into four categories: membrane proteins, metabolism, cytoskeleton, and transcription & translation. The URE3 associated transcripts are shown in Table 2.

A gene was assigned to the membrane encoding group on the basis of the annotated GO term, the presence of a signal peptide, a GPI-anchor signal, or transmembrane domain. The majority of the membrane gene promoters contained a URE3 matrix (73% p<0.0001).

**Figure 5:**
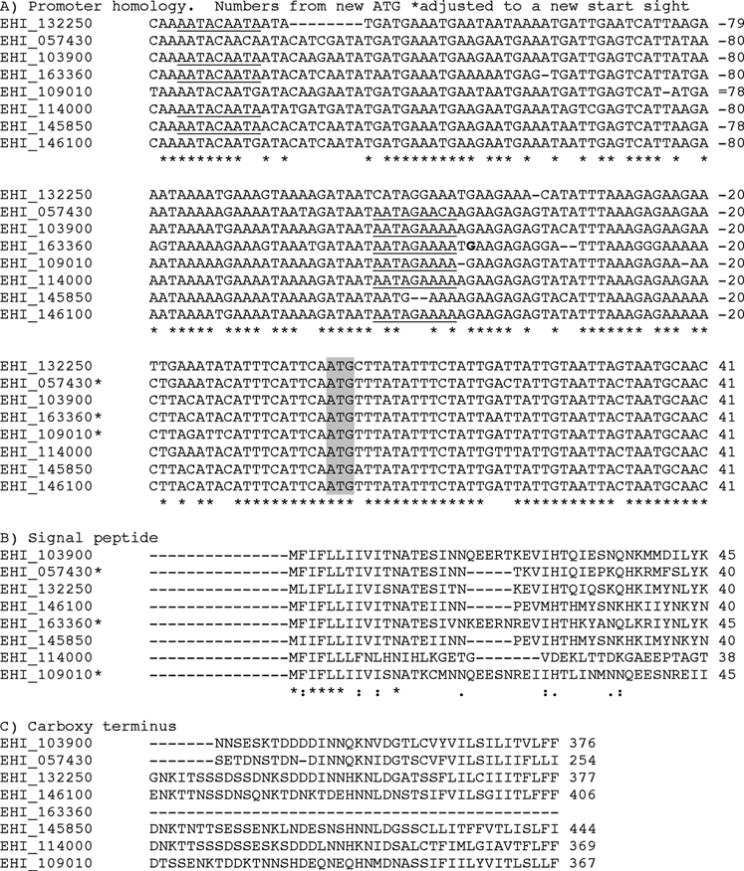
Modulated Transcripts Encoding Potential Membrane Proteins. Sequences were clustered using the ClustalW program. A) Promoter sequences B) Protein sequence at the amino terminal C) Protein sequence at the carboxyl terminal. Numbering is from initial methionine codon or amino acid. * open reading frames where an alternative from the database initiating methionine was used. Potential URE3-BP binding sites are underlined.

The encoded membrane proteins were quite distinct at the protein level. However, a subgroup of these proteins had highly similar promoter, and amino- and carboxyl-terminal sequences (sites of signal peptide and transmembrane domains) (Figure 5). With one exception (EHI_163360), the predicted sizes, pI, and length of the proteins were also quite similar (molecular mass between 29 to 47 kDa, and pI 4.3 to 5.5). In addition, all these proteins contained a hydrophobic domain at the carboxyl terminus, and an anterior potential GPI anchor cleavage/addition site [40].

**Figure 6:**
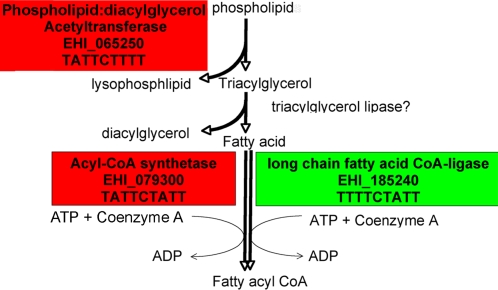
Modulation of Transcripts Encoding Enzymes Involved in Phospholipid Degradation and Fatty Acid Biosynthesis. Transcripts significantly modulated by (EF2)mutURE3-BP are shown in shaded boxes, green indicates a down-regulated transcript and red an up-regulated transcript. As lecithin:cholesterol acyltransferase has homology with phospholipid:diacylglycerol acyltransferase the simpler pathway of triacylglycerol biosynthesis is shown although alternative pathways exist [42],[54]. The locus number and the URE3 motif present within the promoter sequences follow the gene name.

Most of the promoters of the small group of genes encoding metabolic enzymes also contained a URE3 matrix (86% p<0.0001). The enzymes encoded by these genes were linked to phospholipid metabolism. The opposing regulation of two enzymes that catalyze the addition of Coenzyme A to fatty acids (EHI_079300 and EHI_185240) might reflect different substrate specificities of these enzymes [41]. Both could potentially use the fatty acids, which are produced as a consequence of the breakdown of phospholipids by phospholipid:diacylglycerol acyltransferase (PDAT) (Figure 6) [42]. No URE3 matrix was found upstream of the fourth transcript, fatty acid elongase (EHI_092190), which could also be potentially involved in this potential scavenger cell pathway.

### EF(2)mutURE3-BP expression induced migration of amebic trophozoites

**Figure 7:**
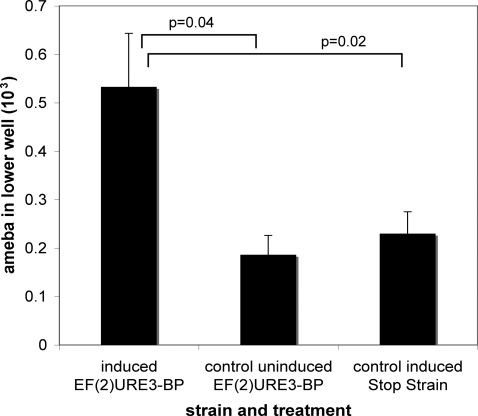
EF(2)mutURE3-BP expression induced migration of amebic trophozoites. Expression of the EF(2)mutURE3-BP and STOP- EF(2)mutURE3-BP transcripts was induced by the addition of tetracycline. Analysis of trophozoite migration was then done by a transwell assay. Data shown are representative of assays using two independently transfected trophozoite lines with the relevant expression vectors in 4 separate experiments. The data are shown as mean ± SEM of the number of cells migrated measured using CellTracker™ Green CMFDA as described in Materials and Methods.

To determine whether URE3-BP regulated the promigratory effects of trophozoites, transwell migration assays were performed as described in materials and methods. A two fold increase in migrating trophozoites was observed when comparing ameba induced to express EF(2)mutURE3-BP to uninduced controls (p = 0.04) or to the induced control stop strain transfected with the construct STOP- EF(2)mutURE3-BP (p = 0.02) (Figure 7). No difference was observed in migration when uninduced or induced STOP- EF(2)mutURE3-BP were compared (data not shown).

## Discussion

In this work the DNA consensus motif recognized by the URE3-BP transcription factor was experimentally defined, and then used to identify a subset of *E. histolytica* transcripts modulated by inducible expression of URE3-BP. URE3-BP had previously been shown to regulate the expression of two virulence factors in the parasite. The current studies provide a more global picture of its role in control of gene expression. The key experimental approach was the inducible expression of a dominant positive URE3-BP mutant and the subsequent identification of uniquely altered transcripts. The majority (42/50) of transcripts were repressed. Over half (54%) of the modulated genes had a URE3 matrix in the promoter region while the other half was comprised of genes presumably downstream of control by URE3.

The URE3 matrix was present in the 5′ sequences of URE3-BP modulated genes involved in fatty acid metabolism and in potential membrane or secreted proteins. The latter suggests that phenotypic changes due to the expression of the dominant positive URE3-BP mRNA could occur most noticeably at the cell surface of *E. histolytica* trophozoites.

URE3-BP regulated genes, which encoded proteins with an N terminal signal peptide, included the potential virulence factor EhCP-A7, a cysteine protease, an asparagine-rich antigenic surface protein ariel [43],[44], a novel lectin-like protein, and a subgroup of genes encoding potential surface proteins which appear to have highly conserved promoters and signal peptides. Most unusually the conservation in this group of potential surface proteins was greater at the DNA rather than the protein level. This may represent a gene duplication followed by functional divergence, or possibly a gene recombination event.

A technical limitation of the gene expression analysis was the inability to measure transcript levels of the *hgl*5 and *fdx1* genes that contain URE3 in their promoters, which cannot be distinguished from highly related gene family members that lack URE3-containing promoters. The *hgl*5 gene belongs to a family of five highly similar genes (up to 99%), and ferredoxin is encoded by two identical ORFs, *fdx1*, and *fdx2* (confirmed by Gilchrist *et al* unpublished data). The presence of the URE3 matrix was not much higher than background in the promoters encoding genes involved in either transcription/translation (25% p = 0.035) or cytoskeletal function (25% p = 0.73).

However while we could not demonstrate changes in the level of the ferredoxin transcript, a URE3 associated Fe-hydrogenase EHI_005060 (EC 1.12.7.2) [45], which may be expected to reduce ferredoxin was statistically significantly up-regulated (over two-fold).

Four of the other six metabolic enzymes identified by inducible expression of URE3-BP could be linked in a phospholipid degradation/ fatty acid assimilation pathway (Figure 6). A potentially rate limiting step in a fatty acid biosynthesis pathway appeared to be closely modulated by opposing regulated acyl-Coenzyme A synthetases (acyl-CoA synthetases). The modulated pathway may be involved in the hydrolysis of phospholipids to form fatty acids and important in modification of the cell membrane lipid content [46]. The inclusion of short chain fatty acids in the *E. histolytica* growth media has no impact on either the URE3-BP transcript or on the genes involved in this pathway, suggesting the lack of feedback inhibition of URE3-BP from the products of this pathway [17].

A limitation of this study was that the microarray analysis measured the steady state mRNA levels and we therefore may have missed changes in newly transcribed RNA, especially for abundant transcripts. Changes occurring in mRNA stability and/or transcript processing may obscure changes occurring at the level of transcription [47],[48]. A second limitation is that the high ‘background’ incidence of the URE3 motif (23%) in the promoters of all *E. histolytica* may indicate that there are other factors not yet identified involved in promoter specific recognition by URE3-BP. Because appreciable levels of wild type URE3-BP were still present, this might have contributed to the failure to observe changes in the roughly 2000 genes with putative URE3-BP binding sites for which no change was seen following induction of EF(2)mutURE3-BP. Because of these issues it is a reasonable conclusion that the 50 changed transcripts are an underestimate of the genes regulated by URE3-BP.

The URE3 matrix was absent in 23 of the regulated promoters. Amebae were harvested at nine hours after the addition of tetracycline and shortly after appreciable induction of recombinant URE3-BP protein (Figure 3B). Therefore it is possible that at this time point URE3-BP regulated transcripts may have in turn induced the expression of a set of secondary-response genes [49]. The URE3 associated EHI_004480 ORF encoding a protein with a basic leucine zipper domain, and the EHI_000780 transcript that encodes a potential chromodomain protein, could act as regulators of a secondary response. Among the modulated non-URE3 associated transcripts are members of the virulence associated EhSTIRP family [3],[50] and cytoskeletal genes suggesting a potential involvement in attachment or motility [19]. The promigratory impact of URE3-BP overexpression shown in Figure 7 supported this correlation however identifying truly co-regulated genes is very difficult with this limited data set [51],[52].

In conclusion, we have identified a group of genes, which appear to be regulated by URE3-BP. These genes and their products may represent a network of interconnected responses to environmental signals. The biological consequences of these changes may impact the ability of the organism to colonize the host, and/or control its invasive behavior.

## Supporting Information

Table S1 (0.04 MB DOC)

Table S2 (7.70 MB XLS)
