# Supplementary material for: Targets of the Entamoeba histolytica Transcription Factor URE3-BP
Source: PLoS Negl Trop Dis. 2008 Aug 27;2(8):e282. doi: 10.1371/journal.pntd.0000282 (PMC2565699; doi:10.1371/journal.pntd.0000282)
Supplement: Table S1 — (0.04 MB DOC) [file pntd.0000282.s001.doc]

Supplemental Table 1

| Gene Annotation | probe set id | qRT-PCR Primers |
| --- | --- | --- |
| URE3-BP | EHI_060740 | ^a^AAAGCGCCGCGATATCCACCAATGCAACCACC  AAAAGATCTAGGGATAGTGTTAAGTAATGGGAACC |
| Recombinant URE3-BP specific primer (myc) | myc-EF(2)mutURE3-BP | ATTTCAGAAGAAGATTTAATGC |
| Lgl1 | EHI_035690 | ATTAGAGGTGGAGCAAACGAGG  ATTCTGGATACCATTCAGTTGGATAAGG |
| PDAT | EHI_065250 | GGGGTTTGGGAAATATCAGC  CAGAAGAATGTCCAGCCAAA |
| Acyl-CoA synthetase | EHI_079300 | AGGAATTCCAAAAGGTGCAG  AAACCAATTCTACCACCACCA |
| Membrane protein | EHI_103900 | TTCCTGTTTGATCGGTTTCA  GGAGGAACAGGAAATGATGG |

^a^ underlined are additional sequence (containing a restriction site) added to the oligonucleotide
